# Fertility preservation in adult male patients with cancer: a systematic review and meta-analysis

**DOI:** 10.1093/hropen/hoae006

**Published:** 2024-01-30

**Authors:** Qing Li, Qiong-Yu Lan, Wen-Bing Zhu, Li-Qing Fan, Chuan Huang

**Affiliations:** Department of Oncology, The Second Affiliated Hospital of Nanchang University, Nanchang, People’s Republic of China; Department of Oncology, The Second Affiliated Hospital of Nanchang University, Nanchang, People’s Republic of China; Human Sperm Bank, Reproductive & Genetic Hospital of CITIC-Xiangya, Changsha, Hunan, People’s Republic of China; The Institute of Reproductive and Stem Cell Engineering, Basic Medicine College, Central South University, Changsha, Hunan, People’s Republic of China; Human Sperm Bank, Reproductive & Genetic Hospital of CITIC-Xiangya, Changsha, Hunan, People’s Republic of China; The Institute of Reproductive and Stem Cell Engineering, Basic Medicine College, Central South University, Changsha, Hunan, People’s Republic of China; Human Sperm Bank, Reproductive & Genetic Hospital of CITIC-Xiangya, Changsha, Hunan, People’s Republic of China; The Institute of Reproductive and Stem Cell Engineering, Basic Medicine College, Central South University, Changsha, Hunan, People’s Republic of China

**Keywords:** male fertility preservation, cancer, sperm cryopreservation, reproductive outcome, meta-analysis, sperm use rate, pregnancy rate, miscarriage rate, delivery rate

## Abstract

**STUDY QUESTION:**

Does sperm cryopreservation serve as a feasible and effective method for preserving fertility in adult male patients with cancer?

**SUMMARY ANSWER:**

Sperm cryopreservation is an effective fertility preservation method and may benefit patients with cancer.

**WHAT IS KNOWN ALREADY:**

Sperm cryopreservation is the only way to efficiently preserve male fertility. It is an important procedure in ART. Recently, due to remarkable advances in cancer treatment, an increasing number of studies have reported the outcomes of sperm cryopreservation in patients with cancer.

**STUDY DESIGN, SIZE, DURATION:**

We conducted an extensive literature search for relevant studies published through to 31 December 2021, in the following databases: CENTRAL, CNKI, Cochrane Systematic Reviews, EMBASE, MEDLINE, PUBMED, and Web of Science. The search terms used were ‘(cryopreservation OR freeze OR freezing OR banking OR cryostorage OR storage) AND (sperm OR semen OR spermatozoon) AND (cancer OR tumor OR malignancy OR neoplasm)’.

**PARTICIPANTS/MATERIALS, SETTING, METHODS:**

We included all studies that reported offering or attempting to cryopreserve sperm before or during cancer treatment in male patients considered at risk of treatment-related fertility impairment. We evaluated the eligibility of all data in each study. The major exclusion criteria were as follows: non-cancer patients; pediatric and adolescent cancer patients; not reporting the use of cryopreserved sperm; use of fresh semen for ART; not reporting the number of patients with cancer offered sperm cryopreservation or attempting to do so before or during treatment; using an experimental fertility preservation technique such as preservation of testicular tissue or spermatogonial stem cells; duplicate data; abstracts, case report, comments, reviews, or editorials; insufficient data reported. The quality of the included studies was assessed using the Newcastle–Ottawa scale and the Methodological Index for Non-Randomized Studies.

**MAIN RESULTS AND THE ROLE OF CHANCE:**

This meta-analysis included 69 non-randomized studies, with 32 234 patients referred for sperm analysis and 23 178 patients cryopreserving at least one sperm sample. The pooled failed-to-cryopreserve rate was 10% (95% CI, 8–12%), and the sperm disposal and sperm use rates were 23% (95% CI, 16–30%) and 9% (95% CI, 8–10%), respectively. The pregnancy, miscarriage, and delivery rates were 28% (95% CI, 22–33%), 13% (95% CI, 10–17%), and 20% (95% CI, 15–25%), respectively. Subgroup analysis showed higher pregnancy and delivery rates, as well as a lower failed-to-cryopreserve rate, in recent studies compared to those released a decade ago. The studies from Asia reported higher sperm disposal and pregnancy rates than in other continents. Our analysis showed clinical pregnancy rates per cycle of 34% (27–41%), 24% (14–35%), and 9% (5–15%) and delivery rates per cycle of 23% (17–30%), 18% (11–26%), and 5% (1–9%) for ICSI, IVF, and IUI, respectively.

**LIMITATIONS, REASONS FOR CAUTION:**

As with all meta-analyses, some limitations should be considered. The first limitation of our study is that the data span 36 years. During this time, the World Health Organization has revised its sperm analysis standards, and other important changes have been made. There is also a limitation in that the outcome does not analyze the correlation between the type of cancer and sperm quality. Many of the earlier studies were limited by small sample sizes and a lack of control groups. Furthermore, almost all studies did not consider the severity of the disease, which could potentially have a substantial impact on the results. Consequently, further research should evaluate the effect of the type of cancer and, in particular, the severity of the condition on sperm quality in order to draw more precise conclusions. Similarly, it is inappropriate that most studies failed to differentiate between patients with different types of tumors and instead drew generalized conclusions that are presumed to apply to all patients with cancer. In the present analysis, we did not have in-depth information on patients’ disease, and although extensive efforts were made to conduct a thorough systematic review and meta-analysis of the outcomes for patients with various types of tumors, the results must be acknowledged as being subject to bias. However, the use of average results obtained in each study, without the patient-level data, might also represent a source of bias.

**WIDER IMPLICATIONS OF THE FINDINGS:**

Sperm cryopreservation is an effective fertility preservation method and may benefit patients with cancer. The observed utilization rate of frozen sperm at 9% may underestimate the actual usage, as the short follow-up period is inadequate for obtaining comprehensive data on the use of frozen sperm in young cancer survivors. ART plays an important role in fertility preservation and the achievement of pregnancy, with this meta-analysis showing that ICSI delivers better clinical outcomes than IVF or IUI in patients with cancer undergoing fertility preservation.

**STUDY FUNDING/COMPETING INTERESTS:**

This work was supported by the National Natural Science Foundation of China (grant no. 82001634, 81960550), and the China Postdoctoral Science Foundation (2019M661521). There are no competing interests to declare.

**REGISTRATION NUMBER:**

CRID 42022314460.

WHAT DOES THIS MEAN FOR PATIENTS? The increasing number of cancer survivors is attributed to advances in cancer treatment. However, these treatments can have adverse effects on male fertility, either temporarily or permanently. For adult males undergoing treatment for cancer who are concerned about their future fertility, sperm cryopreservation (storing sperm at a very low temperature) is the most effective method for preserving fertility. This procedure is integral to assisted reproductive technologies (e.g. IVF) and should be initiated before the onset of fertility-compromising oncological procedures. Therefore, our study aimed to evaluate the effectiveness of sperm cryopreservation in preserving fertility and reproductive outcomes in male cancer patients, with a particular emphasis on the effects of various assisted reproductive technology methods on reproductive outcomes when using cryopreserved sperm. Encompassing 69 studies and 32 234 patients referred for sperm analysis, as well as 23 178 patients cryopreserving at least one sperm sample, the results provide further support for the effectiveness of sperm cryopreservation as a method of fertility preservation in male cancer patients. Despite the fact that only 9% of frozen sperm is currently being utilized, the limited duration of follow-up does not provide enough data to draw conclusions about the use of frozen sperm in young cancer survivors. These preserved samples have been used successfully in various assisted reproductive technology procedures to achieve pregnancy, with intracytoplasmic sperm injection (ICSI) demonstrating better clinical outcomes compared to IVF and intrauterine insemination. Therefore, it is recommended that male cancer patients seeking to preserve their future fertility discuss sperm cryopreservation with their healthcare providers before commencing treatment, as this measure could be pivotal in preserving their ability to conceive after treatment.

## Introduction

According to the World Health Organization (WHO), infertility has become the third most common disease affecting human life and health, after cardiovascular disease and cancer (Rhoton-Vlasak, 2000). Studies have reported that the distress related to infertility is more prevalent among male cancer survivors than unaffected males (Jacobs and Vaughn, 2012; Hibi *et al.*, 2020). The number of cancer survivors has been increasing with recent improvements in cancer treatment modalities. However, cancer treatments, including surgery, radiotherapy, and chemotherapy, can have a transitory or permanent detrimental impact on male fertility. Their gonadotoxic side effects can severely impair fertility in an agent- and dose-dependent way (Del-Pozo-Lerida *et al.*, 2019; Appiah, 2020), and combination treatments of radiotherapy and chemotherapy are more gonadotoxic than either modality alone (Vakalopoulos *et al.*, 2015). Hence, a decline in fertility potential brought about by cancer diagnosis/treatment has one of the biggest impacts on the long-term quality of life of patients with cancer (Behboudi-Gandevani *et al.*, 2021).

Sperm cryopreservation is the only way to efficiently preserve male fertility. It is an important procedure in ART. The importance of consistently addressing the issue of fertility preservation in the course of cancer diagnosis and treatment has been stressed by organizations such as the American Society for Reproductive Medicine (ASRM) (Practice Committee of the American Society for Reproductive Medicine, 2019) and the American Society of Clinical Oncology (ASCO) (Oktay *et al.*, 2018) who have issued formal recommendations urging clinicians to inform their patients about the potential impact of cancer treatments on fertility and offer solutions for fertility preservation, including sperm cryopreservation when necessary. Nevertheless, it has been reported that the utilization rate of stored sperm remains low among male patients with cancer (van Casteren *et al.*, 2008; Ferrari *et al.*, 2016). The utilization rate of cryopreserved sperm is often under 10% and differs widely among studies, as was recently summarized (Ferrari *et al.*, 2016). Recently, owing to remarkable advances in ART, an increasing number of studies have reported the outcomes of sperm cryopreservation in patients with cancer (Grin *et al.*, 2021).

We performed this systematic review and meta-analysis to summarize the current evidence on sperm cryopreservation and reproductive outcomes in adult male patients with cancer, including the most recent contributions and particularly emphasizing the impact of different ART procedures on the reproductive outcomes when using sperm cryopreserved before starting cancer treatment. The main objective of the study was to assess whether sperm cryopreservation serves as a feasible and effective method for preserving fertility in adult male patients with cancer.

## Methods

### Literature search

This study was registered in the International Prospective Register of Systematic Reviews and Meta-analysis Protocols registry (PROSPERO; registration number: CRID 42022314460). We conducted this systematic review and meta-analysis following the Preferred Reporting Items for Systematic Review and Meta-Analysis Protocols (PRISMA-P) (Moher *et al.*, 2015; Shamseer *et al.*, 2015). Briefly, we searched the CENTRAL, Cochrane systematic reviews, EMBASE, MEDLINE, PUBMED, and Web of Science databases using the following terms: ‘(cryopreservation OR freeze OR freezing OR banking OR cryostorage OR storage) AND (sperm OR semen OR spermatozoon) AND (cancer OR tumor OR malignancy OR neoplasm)’. The last search update was on 31 December 2021. Filters were applied for English language, study type (to exclude reviews and case reports), studies conducted on humans, and male sex. The most recent or complete study was included in case of overlapping or duplicated data from the same researchers.

### Selection criteria and outcomes of interest

All studies reporting on offering or attempting to cryopreserve sperm before or during cancer treatment in male patients considered at risk of treatment-related fertility impairment were included. We evaluated the eligibility of all data in each study. The major exclusion criteria were as follows: non-cancer patients; pediatric and adolescent cancer patients; not reporting the use of cryopreserved sperm; use of fresh semen for ART; not reporting the number of patients with cancer offered sperm cryopreservation or attempting to do so before or during treatment; using an experimental fertility preservation technique such as preservation of testicular tissue or spermatogonial stem cells; duplicating data; abstracts, case report, comments, reviews, or editorials; insufficient data reported (i.e. impossible to extract primary data).

Two investigators (QL and CH) established the inclusion and exclusion criteria. The retrieved studies were initially screened for potential eligibility based on the title, abstract, and content. Disagreements about study eligibility were resolved by discussion with a third author (Z.W.B.).

### Data extraction

Two investigators (QL and CH) extracted the data from all included studies. Cases of disagreement were resolved by discussion to reach consensus. We developed a standardized data extraction sheet and extracted the baseline clinical and demographic characteristics from the studies. We recorded the following information from each study: name of the first author; publication year; country; years of follow-up; age at sperm storage; cancer type; failed-to-cryopreserve rate (number of failed-to-cryopreserve patients divided by the number of patients attempting cryopreservation); sperm disposal rate (number of patients who requested the disposal of their cryopreserved sperm for reasons other than unsuccessful cryopreservation divided by the number of patients with successfully cryopreserved samples), sperm use rate (number of patients whose cryopreserved sperm was used for ART divided by the number of patients with successfully cryopreserved samples), pregnancy rate (number of pregnancies divided by the number of ART cycles), miscarriage rate (number of miscarriages divided by the number of ART cycles); delivery rate (number of deliveries divided by the number of ART cycles).

### Assessment of study quality

The quality of the included studies was assessed using the Newcastle–Ottawa scale (Lo *et al.*, 2014) and the Methodological Index for Non-Randomized Studies (Slim *et al.*, 2003). The scale consists of 10 items: a clearly stated aim; a clearly defined study population; representativeness of the sample; report of excluded patients; ascertainment of exposure; prospective collection of data; presence of the outcome of interest; adequate assessment of the outcome of interest; loss to follow-up; and prospective calculation of the study size. The items are scored with one point if affirmative and zero points if negative. The maximum possible total in the scale is 10, the global ideal score being 10 points, representing studies of high quality (Supplementary Table S1). Two reviewers independently scored all studies. Disagreements were resolved by consensus.

### Statistical analysis

The pooled data for cryopreservation of sperm and reproductive outcomes were used to calculate the failed-to-cryopreserve, sperm disposal, sperm use, pregnancy, miscarriage, and delivery rates. Pooling for meta-analysis was performed using the inverse variance method for calculating weights. Random-effects meta-analysis of single proportions was performed to obtain the overall proportions (Suh and Park, 2016). CIs were calculated using the Clopper–Pearson interval for individual studies. Double arcsine transform was performed in studies with data values of 0 or 100%, and the calculated effects were then back-transformed to proportions. Heterogeneity was assessed using Cochran’s Q test (*P *<* *0.05 indicated significant heterogeneity) and the Higgins’ inconsistency index (*I*^2^; >50% indicating significant heterogeneity). Sensitivity analysis was conducted by removing one study at a time to evaluate the quality and consistency of the meta-analysis results. Subgroup analyses within the reproductive outcome cohorts (failed-to-cryopreserve, sperm disposal, sperm use, pregnancy, miscarriage, and delivery rates) included the following covariates: continent (Europe, North America, Oceania, and Asia); and publication year (1982–1991, 1992–2001, 2002–2011, 2012–2021). Furthermore, subgroup analyses were performed within the sperm use, pregnancy, miscarriage, and delivery cohorts for various ART approaches (IUI, IVF, and ICSI). All statistical analyses were conducted using Stata, Version 14.0 (Stata Corporation, College Station, TX, USA).

## Results

### Literature search and selection

**Figure 1.**
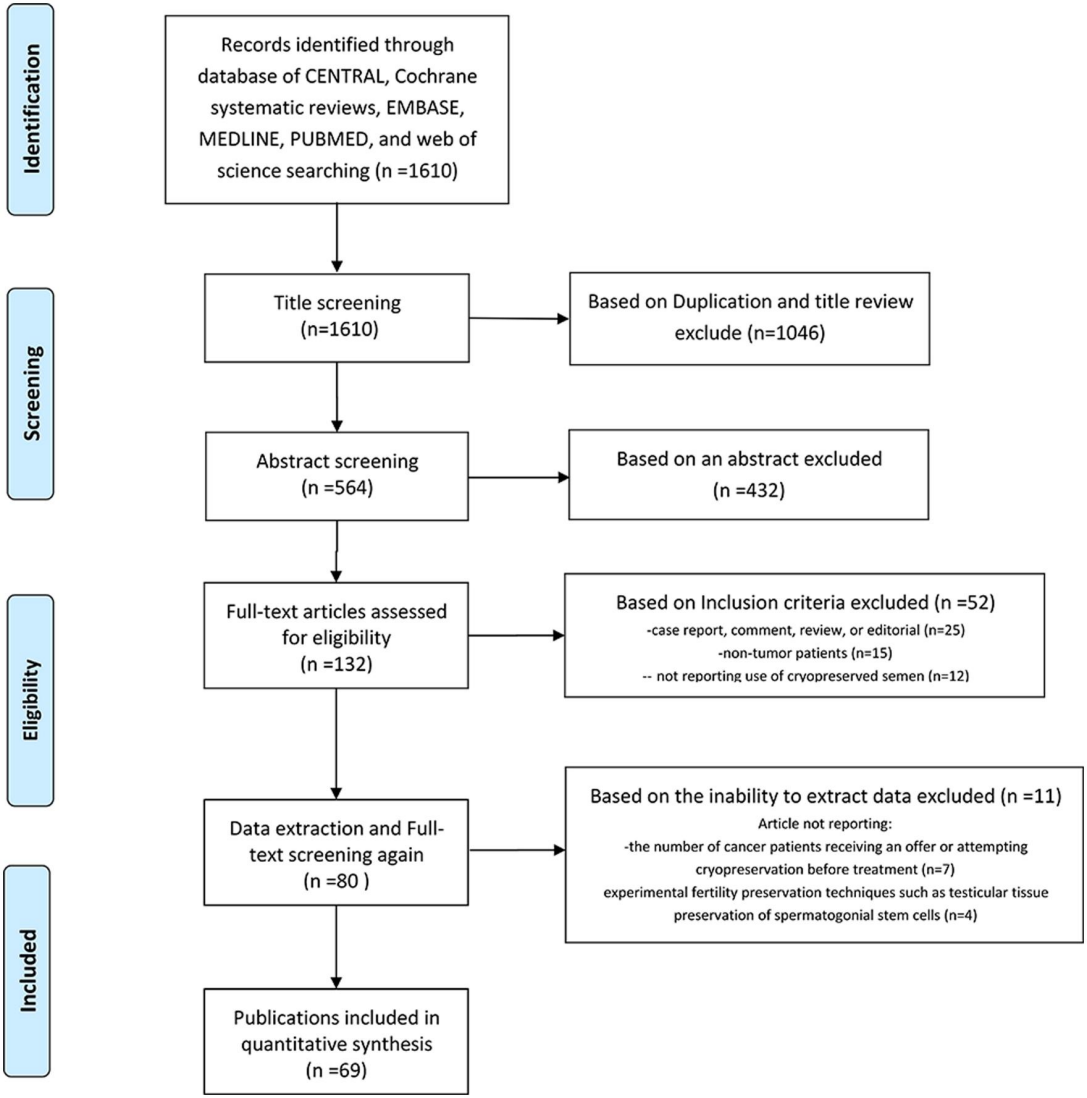
**Flowchart of the study screening and selection procedure for a systematic review and meta-analysis of outcomes following fertility preservation in adult male patients with cancer**.

We retrieved 1610 relevant articles. After removing duplicates and screening the titles, 564 articles remained. After reading the abstracts of these articles, we excluded 432 articles. We evaluated the remaining 132 articles based on the full text and selected 80 non-randomized studies for the systematic review. We excluded 11 of these 80 studies, seven because it was impossible to extract primary data, and four for using experimental fertility preservation techniques. Finally, 69 non-randomized studies were included in the meta-analysis (Fig. 1) (Rhodes *et al.*, 1985; Scammell *et al.*, 1985; Reed *et al.*, 1986; Redman *et al.*, 1987; Fossa *et al.*, 1989; Milligan *et al.*, 1989; Tournaye *et al.*, 1991; Khalifa *et al.*, 1992; Lass *et al.*, 1998; Fitoussi *et al*., 2000; Keane *et al.*, 2000; Ginsburg *et al.*, 2001; Kelleher *et al.*, 2001; Blackhall *et al.*, 2002; Ragni *et al.*, 2003; Spermon *et al.*, 2003; Agarwal *et al.*, 2004; Chung *et al.*, 2004, 2013; Schmidt *et al.*, 2004; Brydøy *et al.*, 2005; Magelssen *et al.*, 2005; Revel *et al.*, 2005; Chang *et al.*, 2006; Meseguer *et al.*, 2006; Zorn *et al.*, 2006; Girasole *et al.*, 2007; Ishikawa *et al.*, 2007; Knoester *et al.*, 2007; Neal *et al.*, 2007; Hourvitz *et al.*, 2008; van Casteren *et al.*, 2008; Crha *et al.*, 2009; Selk *et al.*, 2009; Ping *et al.*, 2010, 2014; Babb *et al.*, 2012; Bizet *et al.*, 2012; Freour *et al.*, 2012; Keene *et al.*, 2012; Sheth *et al.*, 2012; Botchan *et al.*, 2013; Johnson *et al.*, 2013; Dearing *et al.*, 2014; Greaves *et al.*, 2014; van der Kaaij *et al.*, 2014; Záková *et al.*, 2014; García *et al.*, 2015; Sonnenburg *et al.*, 2015; Tomlinson *et al.*, 2015; Depalo *et al.*, 2016; Muller *et al.*, 2016; Kobayashi *et al.*, 2017; Hamano *et al.*, 2018; Levi-Setti *et al.*, 2018; Machen *et al.*, 2018; Negoro *et al.*, 2018; Ukita *et al.*, 2018; Fu *et al.*, 2019; Noetzli *et al.*, 2019; Song *et al.*, 2019; Uçar *et al.*, 2019; Ito *et al.*, 2020; Ferrari *et al.*, 2021; Lackamp *et al.*, 2021; Liu *et al.*, 2021; Papler *et al.*, 2021; Stigliani *et al.*, 2021; Yamashita *et al.*, 2021). These studies included 32 234 patients referred for sperm analysis and 23 178 whose sperm was cryopreserved at least once. Patient age at storage was 16–51 years. Sixty-five studies reported on mixed tumor types. A total of 91.3% (63/69) studies focused on testicular cancer, leukemia, and lymphoma.

### Characteristics of the included studies

The characteristics of the 69 included studies are presented in Table 1. All but nine studies have been published since 2000. Of these 60 studies, six were published in 2021. The studies were conducted in over 23 countries; 13 in the USA, eight in the UK, seven in Japan, six in China, six in Italy, four in France, and four in The Netherlands. Canada, Norway, and Israel recorded three articles each, while the remaining countries recorded 1 or 2 articles each. Among the studies, 62 reported sperm use, and 34 reported pregnancy outcomes. We evaluated the ART approaches in studies reporting pregnancy outcomes. As shown in Table 2, we conducted a systematic review and meta-analyses on 19 studies reporting IUI cycles, 16 reporting IVF cycles, and 22 reporting ICSI cycles. In most cases, more than one method was reported per study.

**Table 1.** Characteristics of the studies included in a meta-analysis of ART outcomes following fertility preservation in adult male patients with cancer.

Studies	Country	Number of patients	Date of study	Follow-up years	Age at storage	Cancer type	Failed-to-cryopreserve	Sperm discarded rate	Sperm used rate	Pregnancy rate	Miscarriage rate	Live births rate
Rhodes *et al.* (1985)	USA	24	1975–1984	9	–	Hodgkin’s and non-Hodgkin’s lymphoma, testicular cancer, various hematologic or bone/connective tissue tumors	–	–	16.7% (4/24)	–	–	–
Scammell *et al.* (1985)	UK	22	1976–1984	8	–	Hodgkin’s disease and testicular tumor	–	–	–	7.0% (8/115)	12.5% (1/8)	6.1% (7/115)
Reed *et al.* (1986)	USA	54	–	–	–	Testicular carcinoma or lymphoma	–	25.9% (14/54)	13.0% (7/54)	–	–	–
Redman *et al.* (1987)	USA	71	1975–1982	7	–	Hodgkin’s disease	–	–	15.5% (11/71)	9.6% (7/73)	–	4.1% (3/73)
Milligan *et al.* (1989)	UK	2219	1977–1987	8	–	–	–	–	6.0% (133/2219)	–	–	–
Fossa *et al.* (1989)	Norway	147	1979–1987	6	–	Testicular cancer	41.8% (38/91)	–	7.5% (4/53)	–	–	–
Tournaye *et al.* (1991)	Belgium	11	1985–1991	4	23	Hodgkin’s disease	–	–	45.5% (5/11)	25.9% (7/27)	–	22.2% (6/27)
Khalifa *et al.* (1992)	USA	10	1986–1990	4	33.4	Seminoma, testicular carcinoma, leiomyosarcoma of the prostate, Wegener’s granulomatosis, non-Hodgkin’s, and Hodgkin’s lymphoma.	–	–	–	40% (4/10)	25% (1/4)	30% (3/10)
Lass *et al.* (1998)	UK	231	1989–1997	8	28	Testicular tumors, hematological malignancy, other cancer	17.3% (40/231)	–	3.1% (6/191)	33.3% (5/15)	–	20% (3/15)*
Keane *et al.* (2000)	Ireland	58	1998	–	30	Testicular carcinoma, lymphoma, leukemia, and other	0% (0/58)	1.7% (1/58)	3.4% (2/58)	–	–	–
Fitoussi *et al.* (2000)	France	316	1976–1996	20	27.5	Hodgkin’s disease	–	–	13.8% (13/94)	10.2% (9/88)	11.1% (1/9)	2.3% (2/88)
Ginsburg *et al.* (2001)	USA	19	–	–	31	Hodgkin’s lymphoma, CML, brain tumor, testicular cancer, thyroid cancer, melamoma	–	–	–	–	21.4% (3/14)	27.5%(11/40)
Kelleher *et al.* (2001)	Australia	930	–	–	–	–	10.4% (97/930)	37.6% (313/833)	8.2% (68/833)	34.1% (29/85)	–	–
Blackhall *et al.* (2002)	UK	122	1978–1990	12	24	Hodgkin’s disease	5.7% (7/122)	25.2% (29/115)	28.7% (33/115)	–	–	–
Ragni *et al.* (2003)	Italy	776	1986–2001	15	28	Testicular tumors, lymphoma, Leukemias, other	11.6% (90/776)	18.1% (124/686)	5.2% (36/686)	15.9% (14/88)	14.3% (2/14)	13.6% (12/88)
Spermon *et al.* (2003)	The Netherlands	99	1982–1999	18	–	Testicular tumors	21.2% (21/99)	–	16.7% (13/78)	–	–	–
Agarwal *et al.* (2004)	USA	318	1982–2001	19	30	Testicular cancer, Hodgkin’s disease, and other	–	–	9.1% (29/318)	18.4% (16/87)	–	13.8% (12/87)
Chung *et al.* (2004)	USA	164	1993–2003	10	29.5	Testicular cancer, Hodgkin’s lymphoma, leukemia, and gastrointestinal cancers and other	–	19.5% (32/164)	4.7% (6/127)	10% (2/20)	–	10% (2/20)
Schmidt *et al.* (2004)	Denmark	67	1996–2003	7	–	Testicular cancer, lymphomas	17.9% (12/67)	–	–	31.5% (23/73)	–	24.7% (18/73)
Revel *et al.* (2005)	Israel	21	1999–2002	3	33	Hodgkin’s lymphoma, non-Hodgkin’s lymphoma, sarcoma, seminoma, testicular teratoma, inguinal hystiocytoma, prostate carcinoma, and acute lymphocytic leukemia.	–	–	–	41.9% (26/62)	30.7% (8/26)	29.0% (18/62)
Zorn *et al.* (2006)	Slovenia	360	1996–2003	7	31.6	Testicular cancer, Hodgkin’s disease, non-Hodgkin lymphoma, leukemia, and other	–	–	5.6% (20/360)	22.6% (7/31)	28.6% (2/7)	12.9% (4/31)
Magelssen *et al.* (2005)	Norway	422	1983–2002	19	–	Testicular cancer	–	–	6.9% (29/422)	–	–	–
Brydøy *et al.* (2005)	Norway	326	1998–2002	4	–	Testicular cancer	–	–	5.5% (18/326)	–	–	–
Girasole *et al.* (2007)	USA	129	1994–2004	10	26.2	Testicular carcinoma	–	–	6.5% (2/31)	–	–	–
Chang *et al.* (2006)	China	75	1995–2004	9	25.7	Leukemia, lymphoma, testicular cancer, and other	–	17.3% (13/75)	4.0% (3/75)	0% (0/5)	–	0% (0/5)
Meseguer *et al.* (2006)	Spain	186	1991–2004	13	27.1	Testicular cancer, Hodgkin’s lymphoma, and other	–	8.6% (16/186)	16.1% (30/186)	45.7% (16/35)	–	34.3% (12/35)
Knoester *et al.* (2007)	USA	8	2002–2005	3	50.1	Prostate cancer	–	–	12.5% (1/8)	–	–	–
Ishikawa *et al.* (2007)	Japan	130	2002–2005	3	30.1	Testicular cancer/lymphoma/leukemia/other	9.2% (12/130)	–	3.4% (4/118)	42.9% (3/7)	–	42.9% (3/7)
Neal *et al.* (2007)	Canada	146	1995–2005	10	–	Hodgkin lymphoma, testicular cancer	–	15.1% (22/146)	14.4% (21/146)	–	–	–
Hourvitz *et al.* (2008)	Israel	118	1994–2005	11	38.5	Testicular cancer, lymphomas, and prostate cancer and other	–	–	–	56.8% (96/169)	11.5% (11/96)	50.3% (85/169)
van Casteren *et al.* (2008)	The Netherlands	629	1983–2004	21	27	Testicular germ cell tumors, hematological malignancies, extragonadal germ cell tumors, one melanoma, and two schwannomas	11.4% (72/629)	5.2% (29/557)	7.5% (42/557)	26.7% (27/101)	7.4% (2/27)	21.8% (22/101)
Selk *et al.* (2009)	Canada	388	2002–2005	3	–	Testicular cancer and Hodgkin lymphoma and other	5.4% (21/388)	–	8.4% (31/367)	33.3% (16/48)	–	–
Crha *et al.* (2009)	Czech Republic	619	1995–2006	11	26.2	Malignant testicular cancer, malignant neoplasms of the lymphatic and hematopoietic tissues, and other	–	–	16.6% (28/619)	–	–	–
Ping *et al.* (2010)	China	30	2003–2008	5	–	–	–	–	6.7% (2/30)	50% (1/2)	–	50% (1/2)
Freour *et al.* (2012)	France	1042	1997–2007	10	28.58	Testicular cancer/lymphoma/leukemia/other	5.3% (55/1042)	–	8.3% (82/987)	19.1% (34/178)	14.6% (26/178)	–
Babb *et al.* (2012)	UK	112	1979–2007	28	33	Hematological malignancy	–	–	59.5% (25/42)	–	–	–
Keene *et al.* (2012)	UK	180	1995–2009	14	16.1	Lymphoma, leukemia, bone tumors, testicular tumors, soft tissue sarcoma, brain tumor, germ cell tumors, and other cancers	22.7% (35/154)	16.8% (20/119)	0.8% (1/119)	50% (1/2)	–	–
Sheth *et al.* (2012)	USA	4881	2002–2010	18	–	–	6.4% (17/266)	14.9% (37/249)	8.4% (21/249)	–	–	–
Bizet *et al.* (2012)	France	1007	1995–2009	14	29.3	Testicular cancer, lymphoma, other hematological cancers or other types of cancer	9.5% (96/1007)	18.7% (170/911)	6.3% (57/911)	25.6% (30/117)		19.1% (22/115)
Botchan *et al.* (2013)	Israel	682	–	20	31	Testicular cancer , lymphoma, and other types of cancer	–	–	10.3% (170/682)	21.7% (40/184)	9.3% (4/43)	16.3% (30/184)
Chung *et al.* (2013)	China	130	1995–2012	17	27	Testicular cancer, hematological malignancy, Gastro-intestinal malignancy, Musculoskeletal malignancy, Neurological malignancy, Nasopharyngeal malignancy, and other	12.0% (15/125)	37.3% (41/110)	3.6% (4/110)	–	–	–
van der Kaaij *et al.* (2014)	France, Belgium, Netherlands, Italy, Switzerland	902	1974–2004	30	–	Hodgkin’s lymphoma	–	–	21.5% (78/363)	–	–	–
Johnson *et al.* (2013)	USA	423	1991–2010	19	29.8	Testicular cancer, lymphoma, and leukemia and other	10.6% (45/423)	42.6% (161/378)	9.5% (36/378)	–	–	–
Dearing *et al.* (2014)	UK	4362	1976–2013	37	32	Testicular cancer/lymphoma/leukemia/other	–	–	6.0% (183/3062)	–	–	–
Greaves *et al.* (2014)	UK	91	1957–2006	49	–	Lymphoma/leukemia	–	–	28.6% (26/91)	–	–	–
Záková *et al.* (2014)	Czech Republic	523	1995–2012	17	28.5	Testicular cancer	6.1% (34/557)	–	6.5% (34/523)	34.8% (16/46)	–	13.0% (6/46)
Ping *et al.* (2014)	China	125	1996–2010	14	36.3	Testicular cancer	–	–	23.8% (5/21)	–	–	–
García *et al.* (2015)	Canada	272	2008–2012	4	36.7	Lymphoma, testicular cancer, leukemia, and other malignancies including sarcoma, gastrointestinal, and central nervous system malignancies	–	–	10.7% (29/272)	–	–	–
Tomlinson *et al.* (2015)	Pakistan	823	–	–	–	Testicular cancer/lymphoma/leukemia/other	–	–	5.5% (45/823)	–	–	–
Sonnenburg *et al.* (2015)	USA	200	–		28.4	Germ cell tumor	–	–	18.0% (11/61)	–	–	–
Muller *et al.* (2016)	The Netherlands	942	1983–2013	20	29	Testicular cancer, Hodgkin’s lymphoma, leukemia, non-Hodgkin’s lymphoma, and other	4.7% (44/942)	33.9% (304/898)	10.7% (96/898)	33.0% (95/288)	–	28.0% (81/288)
Depalo *et al.* (2016)	Italy	778	1999–2015	16	29.23	Seminoma of the testis, Hodgkin’s lymphoma, mixed testicular tumors, germ cell tumors, other tumors, hematological tumors, and non-Hodgkin’s lymphoma	7.3% (57/778)	1.0% (7/721)	2.6% (19/721)	25% (5/20)	–	25% (5/20)
Kobayashi *et al.* (2017)	Japan	122	2006–2015	9	33.6	Testicular, hematological, digestive, and other types	–	–	9.8% (12/122)	–	–	–
Machen *et al.* (2018)	USA	271	1988–2015	27	–	–	–	16.2% (44/271)	1.5% (4/271)	25.0% (1/4)	–	–
Negoro *et al.* (2018)	Japan	257	1994–2013	19	–	Germ cell tumor and hematological disorders	–	–	9.7% (25/257)	–	–	–
Hamano *et al.* (2018)	Japan	111	1999–2016	17	29	Hematological malignancies, testicular cancer	–	–	7.2% (8/111)	–	–	–
Noetzli *et al.* (2019)	Switzerland	169	2002–2012	10	29.6	Acute leukemia	15.2% (5/33)	64.3% (18/28)	7.1% (2/28)	–	–	–
Levi-Setti *et al.* (2018)	Italy	213	1998–2017	19	28.6	Hodgkin’s lymphoma, non-Hodgkin’s lymphoma, leukemia, and myelomas	5.1% (8/156)	–	17.9% (28/156)	21.2% (11/52)	9.1% (1/11)	19.2% (10/52)
Ukita *et al.* (2018)	Japan	31	2004–2017	13	–	Testicular cancer, malignant lymphoma, leukemia, and other	–	–	6.5% (2/31)	–	–	–
Fu *et al.* (2019)	China	145	2006–2017	11	29.3	Testicular cancer/lymphoma/leukemia/other	19.4% (13/167)		9.7% (14/145)	51.5% (17/33)	6.1% (2/33)	30.3% (10/33)
Song *et al.* (2019)	Korea	721	1996–2016	20	27	Leukemia, lymphoma, testis cancer, and other	–	40.8% (294/721)	6.1% (44/721)	–	–	–
Ito *et al.* (2020)	Japan	91	1996–2016	20	–	Testicular cancer /extragonadal germ cell tumors)	17.3% (9/52)	30.2% (13/43)	23.3% (10/43)	47.6% (10/21)	9.5% (2/21)	33.3% (7/21)
Uçar *et al.* (2019)	Turkey	110	2000–2016	16	36	Testicular cancer	–	–	4.5% (5/110)	–	–	–
Ferrari *et al.* (2021)	Italy	1524	1986–2009	23	29	Testicular cancer, Hodgkin Lymphoma, non-Hodgkin lymphoma, leukemia, other solid tumors	9.4% (158/1682)	42.5% (648/1524)	9.4% (144/1524)	–	–	–
Lackamp *et al.* (2021)	Germany	91	1994–2017	23	38	Testicular cancer and malignancies of the lymphatic and hematopoietic tissue and other	–	–	17.6% (16/91)	–	–	–
Stigliani *et al.* (2021)	Italy	682	2004–2019	15	–	Leukemia and lymphoma, testicular cancer, and other	7.3% (50/682)	–	7.2% (26/632)	33.3% (15/45)	33.3% (5/15)	28.9% (13/45)
Yamashita *et al.* (2021)	Japan	567	2018–2019	1	–	Testicular cancer	13.1% (20/153)	–	21.1% (28/133)	–	–	–
Papler *et al.* (2021)	Slovenia	70	2004–2018	14	–	Testicular cancer, Hodgkin lymphoma, Leukemia, and other	–	–	–	35.8% (39/109)	25.6% (10/39)	26.6% (29/109)
Liu *et al.* (2021)	China	339	2010–2019	9	26.7	Germ cell tumors, hematological neoplasms, head and neck cancers, thoracic tumors, abdominal tumors, and others	7.7% (26/339)	34.5% (108/313)	4.2% (13/313)	66.7% (10/15)	20.0% (2/10)	33.4% (5/15)

* Not including two ongoing pregnancies.

**Table 2.** Summary of IUI, IVF, and ICSI results in cycles using cryopreserved semen in the 69 studies included in the meta-analysis.

Studies	IUI			IVF			ICSI		
	Pregnancy rate	Miscarriage rate	Live births rate	Pregnancy rate	Miscarriage rate	Live births rate	Pregnancy rate	Miscarriage rate	Live births rate
Redman *et al.* (1987)	7.0% (8/115)	12.5% (1/8)	6.1% (7/115)	–	–	–	–	–	–
Tournaye *et al.* (1991)	–	–	–	15.8% (3/19)	–	10.5% (2/19)	–	–	–
Khalifa *et al.* (1992)	–	–	–	50.0% (4/8)	25% (1/4)	37.5% (3/8)	0% (0/2)	–	0% (0/2)
Lass *et al.* (1998)	100% (2/2)	–	100% (2/2)	11.1% (1/9)	–	11.1% (1/9)	50.0% (2/4)	–	–
Fitoussi *et al.* (2000)	–	–	2.5% (2/80)	–	–	–	0% (0/8)	–	0% (0/8)
Kelleher *et al.* (2001)	31.4% (11/35)	–	–	21.4% (6/28)	–	–	54.5% (12/22)	–	–
Ragni *et al.* (2003)	7.5% (3/40)	–	–	0% (0/6)	–	0% (0/6)	26.2% (11/42)	–	–
Agarwal *et al.* (2004)	7.1% (3/42)	–	7.1% (3/42)	23.1% (6/26)	–	19.2% (5/26)	36.8% (7/19)	–	21.1% (4/19)
Chung *et al.* (2004)	0% (0/12)	–		20.0% (1/5)	–	20.0% (1/5)	33.3% (1/3)	–	33.3% (1/3)
Schmidt *et al.* (2004)	16.7% (4/24)	–	16.7% (4/24)	–	–	–	38.8% (19/49)	–	30.6% (15/49)
Zorn *et al.* (2006)	–	–	–	–	–	–	22.6% (7/31)	28.6% (2/7)	12.9% (4/31)
Chang *et al.* (2006)	0% (0/2)	–	0% (0/2)	–	–	–	0% (0/3)	–	0% (0/3)
Meseguer *et al.* (2006)	20.0% (1/5)	–	0% (0/5)	–	–	–	50.0% (15/30)	–	40.0% (12/30)
Ishikawa *et al.* (2007)	0% (0/1)	–	0% (0/1)	–	–	–	50.0% (3/6)	–	50.0% (3/6)
van Casteren *et al.* (2008)	14.3% (1/7)	0% (0/1)	14.3% (1/7)	25.0% (8/32)	0% (0/8)	25.0% (8/32)	30.2% (16/53)	6.3% (1/16)	28.3% (15/53)
Crha *et al.* (2009)	22.2% (2/9)	–	22.2% (2/9)	–	–	–	–	–	–
Ping *et al.* (2010)	–	–	–	0% (0/1)	–	0% (0/1)	100% (1/1)	–	100% (1/1)
Freour *et al.* (2012)	12.1% (8/66)	–	12.1% (8/66)	–	–	–	–	–	–
Keene *et al.* (2012)	–	–	–	–	–	–	50.0% (1/2)	–	–
Bizet *et al.* (2012)	12.8% (5/39)	–	10.3% (4/39)	28.6% (2/7)	–	28.6% (2/7)	32.4% (23/71)	–	23.2% (16/69)
Botchan *et al.* (2013)	10.0% (8/81)	0% (0/8)	8.6% (7/81)	0% (0/12)	–	0% (0/12)	35.2% (32/91)	12.5% (4/32)	25.3% (23/91)
Záková *et al.* (2014)	50.0% (3/6)	–	16.7% (1/6)	0% (0/2)	–	0% (0/2)	34.2% (13/38)	–	13.2% (5/38)
Muller *et al.* (2016)	13.9% (15/108)	–	13.0% (14/108)	41.8% (33/79)	–	34.2% (27/79)	46.5% (47/101)	–	39/.6% (40/101)
Levi-Setti *et al.* (2018)	–	–	–	–	–	–	21.2% (11/52)	9.1% (1/11)	19.2% (10/52)
Fu *et al.* (2019)	42.9% (6/14)	–	28.6% (4/14)	57.1% (8/14)	–	28.6% (4/14)	60.0% (3/5)	–	40.0% (2/5)
Ito *et al.* (2020)	–	–	–	47.6% (10/21)	20.0% (2/10)	33.3% (7/21)	–	–	–
Liu *et al.* (2021)	0% (0/1)	–	0% (0/1)	60.0% (3/5)	66.7% (2/3)	20.0% (1/5)	77.8% (7/9)	0% (0/7)	44.4% (4/9)

### Overall semen cryopreservation and reproductive outcomes

**Figure 2.**
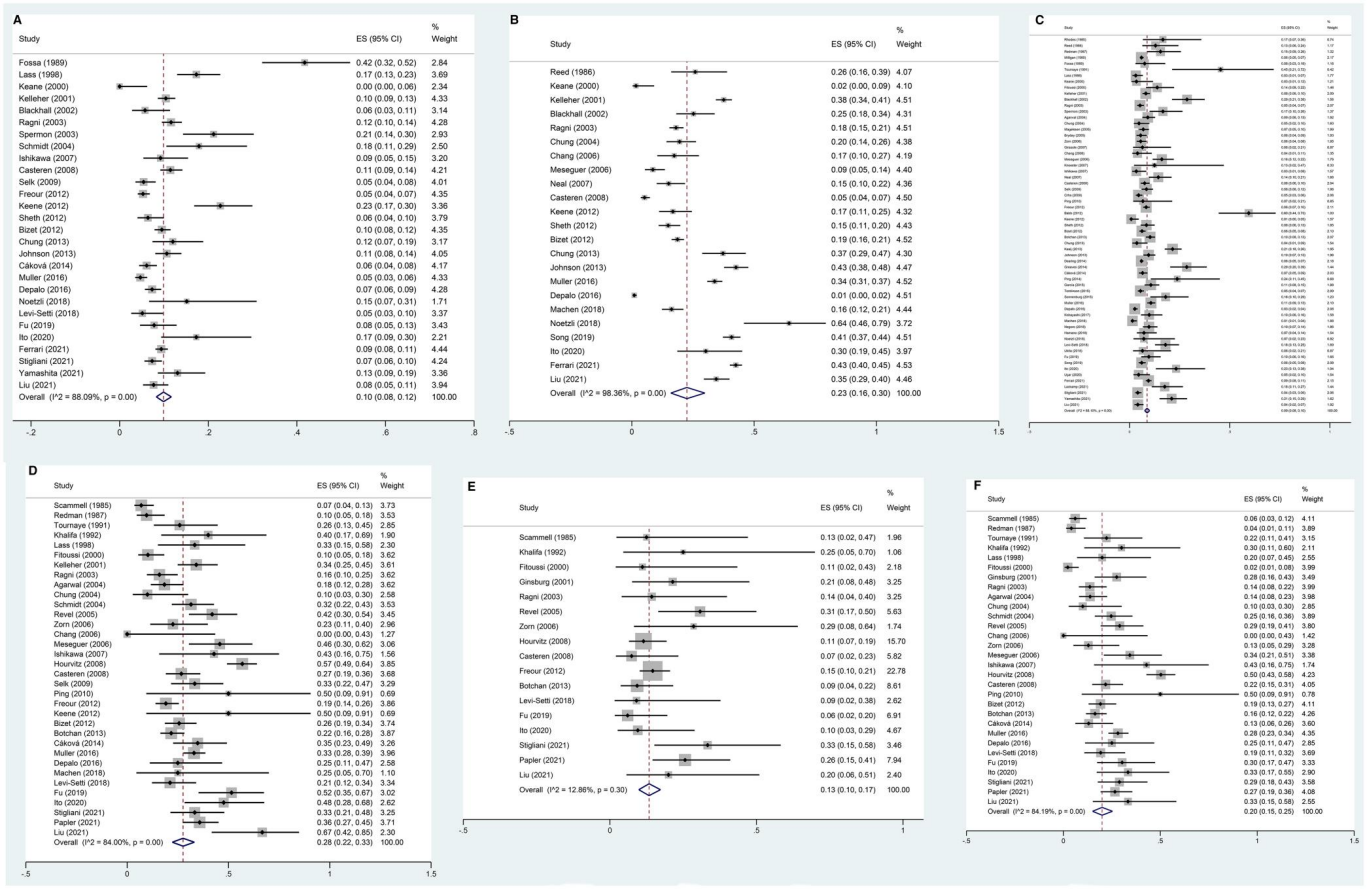
**Forest plot of overall semen cryopreservation in adult male patients with cancer and reproductive outcomes.** Forest plots of: (**A**) the failed-to-cryopreserve rate; (**B**) the disposed sperm rate; (**C**) the sperm use rate; (**D**) the pregnancy rate when using cryopreserved sperm; (**E**) the miscarriage rate after using cryopreserved sperm; and (**F**) the delivery rate after using cryopreserved sperm.

Among the studies that investigated fertility preservation in male patients with cancer, the pooled failed-to-cryopreserve rate was 10% (95% CI, 8–12%). We used a random-effects model as high heterogeneity was found (*P*-value <0.001, *I*^2^=88.1%; Fig. 2A). The pooled sperm disposal rate was 23% (95% CI, 16–30%). We used a random-effects model as high heterogeneity was found (*P *<* *0.001, *I*^2^ = 98.4%; Fig. 2B). The pooled sperm use rate was 9% (95% CI, 8–10%). We used a random-effects model as high heterogeneity was found (*P *<* *0.001, *I*^2^=88.1%; Fig. 2C). The pooled pregnancy rate was 28% (95% CI, 22–33%). We used a random-effects model as high heterogeneity was found (*P *<* *0.001, *I*^2^=84.0%; Fig. 2D). The pooled miscarriage rate was 13% (95% CI, 10–17%), and a random-effects model was used (*P *=* *0.30, *I*^2^=12.9%; Fig. 2E). The pooled delivery rate was 20% (95% CI, 15–25%), and a random-effects model was used (*P *<* *0.001, *I*^2^=84.2%; Fig. 2F).

### Sperm cryopreservation in male patients with cancer

Subgroup analysis of the failed-to-cryopreserve rate by continent found no differences across continents (*P *=* *0.475). However, as shown in Supplementary Fig. S1b, significant differences were noted across publication years (8% (95% CI, 2–17%) vs 11% (95% CI, 8–14%) vs 9% (95% CI, 7–10%), *P *<* *0.001).

The numerically highest sperm disposal rate among continents was in Asia (33% (26–40%)), while the lowest was in Europe (18% (9–30%); Supplementary Fig. S2a). Statistically significant differences across publication years were noted in the sperm disposal rates (34% (95% CI, 31–37%) vs 15% (95% CI, 9–21%) vs 28% (95% CI, 18–39%), *P *<* *0.001).

Similar sperm use rates were noted across continents (*P *=* *0.556) and publication years (*P *=* *0.347; Supplementary Fig. S3).

### Reproductive outcomes in male patients with cancer

**Figure 3.**
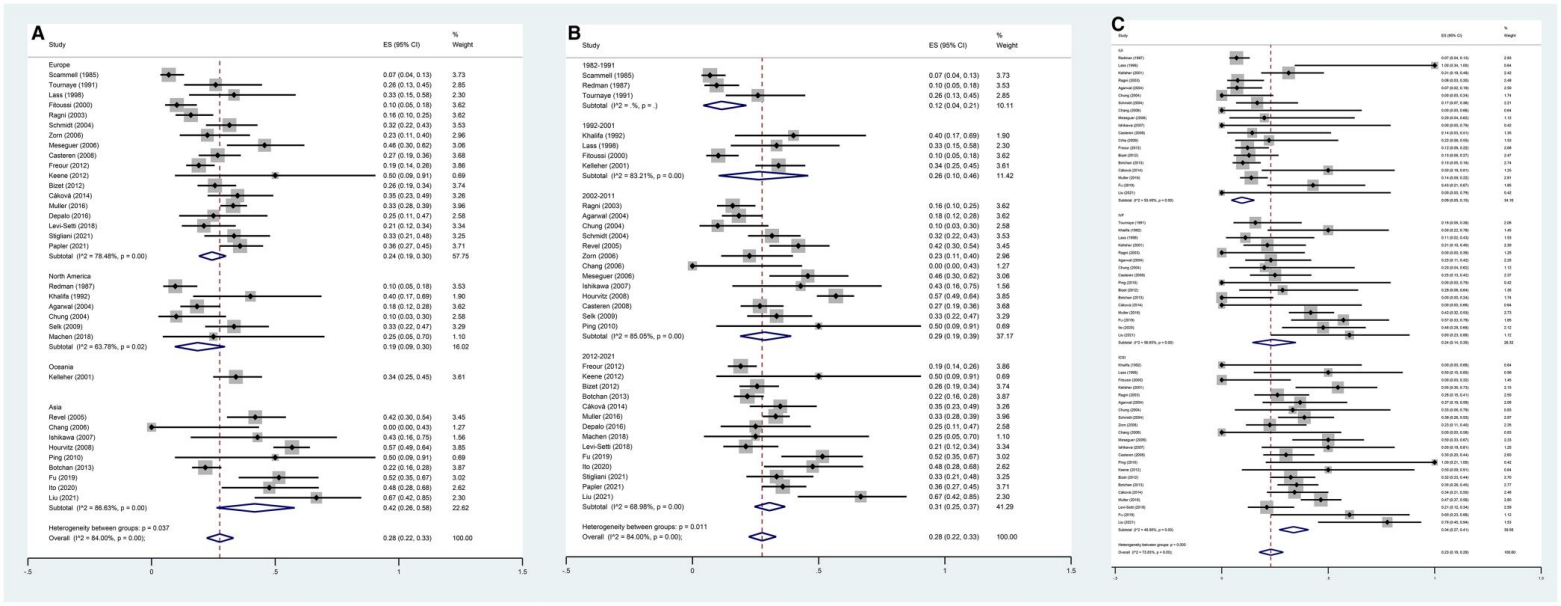
**The subgroup analysis of pregnancy rate in partners of adult male patients with cancer.** Forest plots of: (**A**) the pregnancy rate by continent after using cryopreserved sperm, (**B**) the pregnancy rate by publication year after using cryopreserved sperm, and (**C**) the pregnancy rate by ART method after using cryopreserved sperm.

The numerically highest pregnancy rate among the ART approaches with frozen sperm from patients with cancer was obtained with ICSI (34% (CI, 27–41%)), followed by IVF (24% (CI, 14–35%)) and IUI (9% (CI, 5–15%), Fig. 3C). Subgroup analysis of the pregnancy rate by continent found significant differences among continents (Europe vs North America vs Asia: 24% (CI, 19–30%) vs 19% (CI, 9–30%) vs 42% (CI, 26–58%), *P *<* *0.001) (Fig. 3A) and publication years (12% (CI, 4–21%) vs 26% (CI, 10–46%) vs 29% (CI, 19–39%) vs 31% (CI, 25–37%), *P* = 0.011) (Fig. 3B). Importantly, the more recent the publication year, the higher the reported pregnancy rate.

**Figure 4.**
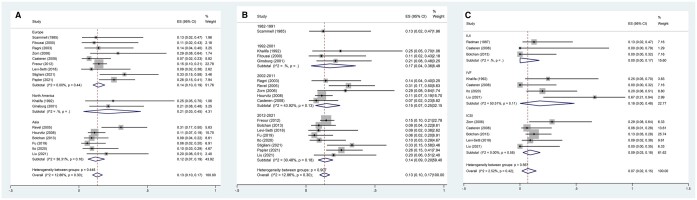
**The subgroup analysis of miscarriage rate in partners of adult male patients with cancer.** Forest plots of: (**A**) the miscarriage rate by continent after using cryopreserved sperm, (**B**) the miscarriage rate by publication year after using cryopreserved sperm, and (**C**) the miscarriage rate by ART method after using cryopreserved sperm.

Similar miscarriage rates were noted among continents (*P *=* *0.448), publication years (*P *=* *0.907), and ART approach (*P *=* *0.567; Fig. 4).

**Figure 5.**
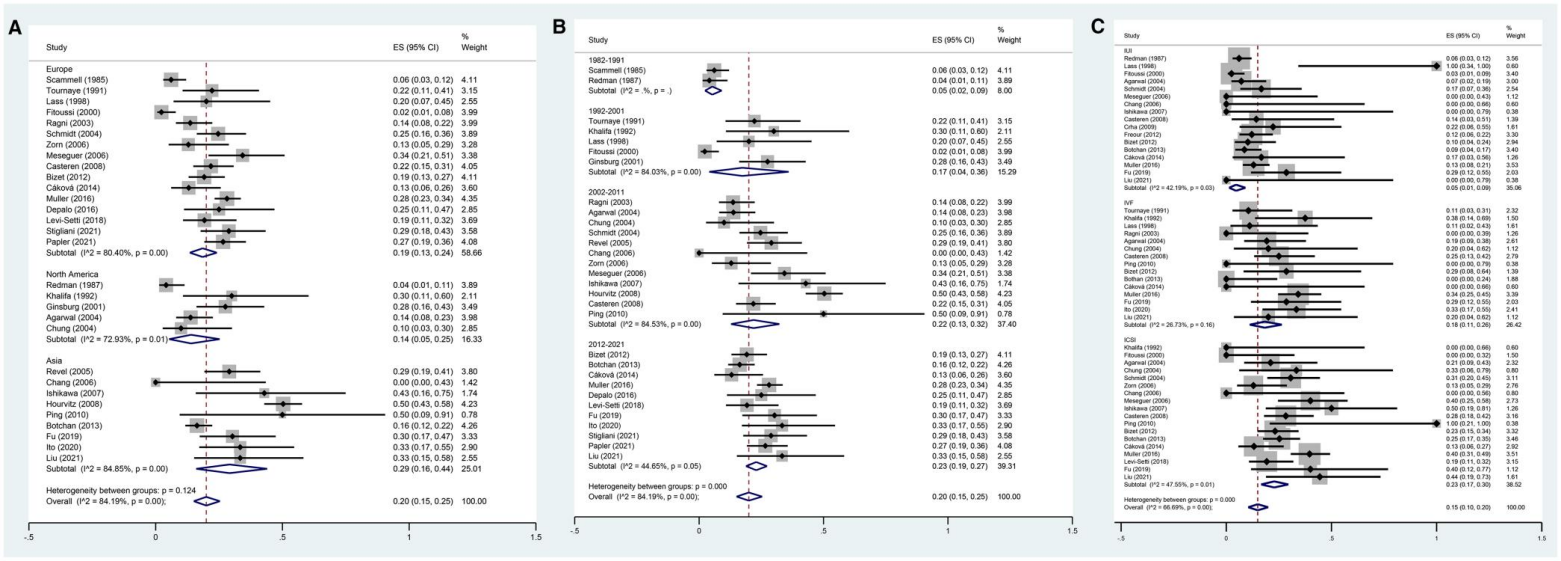
**The subgroup analysis of delivery rate in partners of adult male patients with cancer.** Forest plots of: (**A**) the delivery rate by continent after using cryopreserved sperm, (**B**) the delivery rate by publication year after using cryopreserved sperm, and (**C**) the delivery rate by ART method after using cryopreserved sperm.

The numerically highest delivery rate among the different ART approaches using frozen sperm from patients with cancer was obtained with ICSI (23% (CI, 17–30%)), followed by IVF (18% (CI, 11–26%)) and IUI (5% (CI, 1–9%), Fig. 5C). More recent studies reported higher delivery rates (5% (CI, 2–9%) vs 17% (CI, 4–36%) vs 22% (CI, 13–32%) vs 23% (CI, 19–27%), *P *<* *0.001, Fig. 5B). However, as shown in Fig. 5A, similar delivery rates were noted across continents (*P *=* *0.124).

Meanwhile, as shown in Table 3, the included studies reveal that lymphoma patients experience lower pregnancy (lymphoma vs leukemia vs testicular tumors vs other solid tumors: 17.1% vs 32.6% vs 28.3% vs 33.3%, respectively) and delivery rates (lymphoma vs leukemia vs testicular tumors vs other solid tumors: 10.6% vs 58.8% vs 29.6% vs 35.5%, respectively).

**Table 3.** Pregnancy rate for ART cycles according to type of cancer in the included studies.

Studies	Pregnancy rate					Miscarriage rate					Live births rate				
	Lymphoma		Leukemia	Testicular tumors	Other solid tumors	Lymphoma		Leukemia	Testicular tumors	Other solid tumors	Lymphoma		Leukemia	Testicular tumors	Other solid tumors
	Hodgkin’s	Non-Hodgkin’s				Hodgkin’s	Non-Hodgkin’s				Hodgkin’s	Non-Hodgkin’s			
Redman *et al.* (1987)	9.6% (7/73)	_	_	_	_	_	_	_	_	_	4.1% (3/73)	_	_	_	_
Tournaye *et al.* (1991)	25.9% (7/27)	_	_	_	_	_	_	_	_	_	22.2% (6/27)	_	_	_	_
Lass *et al.* (1998)	18.2% (2/11)	100% （1/1）	100% （1/1）	50% (1/2)	_	_	_	_	_	_	9.1% (1/11)*	100% （1/1）	100% （1/1）	0% (0/2)*	_
Fitoussi *et al.* (2000)	10.2% (9/88)	_	_	_	_	11.1% (1/9)	_	_	_	_	2.3% (2/88)	_	_	_	_
Agarwal *et al.* (2004)	13.5% (5/37)	_	_	18.8% (6/32)	_	_	_	_	_	_	8.1% (3/37)	_	_	12.5% (4/32)	_
Chung *et al.* (2004)	9.1% (1/11)	_	_	12.5% (1/8)	0% (0/1)	_	_	_	_	_	9.1% (1/11)	_	_	12.5% (1/8)	_
Revel *et al.* (2005)	27.8% (5/18)	45.5% (5/11)	100% （1/1）	38.9% (7/18)	57.1% (8/14)	20% (1/5)	40% (2/5)	0% （0/1）	57.1% (4/7)	12.5% (1/8)	22.2% (4/18)	27.3% (3/11)	100% （1/1）	16.7% (3/18)	50% (7/14)
Ishikawa *et al.* (2007)	0% (0/4)		100% (2/2)	100% (1/1)	_	_		_	_	_	0% (0/4)		100% (2/2)	100% (1/1)	_
Hourvitz *et al.* (2008)	_	_	_	57.9% (40/69)	_	_	_	_	_	_	_	_	_	52.2%（36/69）	_
Ping *et al.* (2010)	_	_	_	50% (1/2)	_	_	_	_	_	_	_	_	_	50% (1/2)	_
Freour *et al.* (2012)	18.8% (6/32)		25% (18/72)	11.8% (8/68)	7.1% (2/28)	_		_	_	_	_	_	_	_	_
Botchan *et al.* (2013)	22.2% (16/72)		_	29.6% (16/54)	_	_		_	_	_	18.1% (13/72)		_	29.6% (16/54)	_
Záková *et al.* (2014)	_	_	_	34.8% (16/46)	_	_	_	_	_	_	_	_	_	13.0% (6/46)	_
Fu *et al.* (2019)	66.7% (2/3)		45.5% (5/11)	55.6% (5/9)	50% (5/10)	_		_	_	_	33.3% (1/3)		36.4% (4/11)	22.2% (2/9)	30% (3/10)
Liu *et al.* (2021)	25% (1/4)		100% (2/2)	100% (2/2)	71.4% (5/7)	_		_	_	40.0% (2/5)	0% (0/4)		100% (2/2)	100% (2/2)	14.3% (1/7)
Total	17.1% (67/392)		32.6% (29/89)	28.3% (88/311)	33.3% (20/60)	21.1% (4/19)		0% (0/1)	57.1% (4/7)	23.1% (3/13)	10.6% (38/359)		58.8% (10/17)	29.6% (72/243)	35.5% (11/31)

* Not including one ongoing pregnancy.

### Sensitivity analysis

Sensitivity analysis assessed the failed-to-cryopreserve, sperm disposal, sperm use, pregnancy, miscarriage, and delivery rates (Supplementary Fig. S4). The sensitivity assessment found the results to be stable.

## Discussion

Fertility preservation is the only option for patients with cancer who are hoping to maintain their fertility despite cancer treatments. Advancements in early diagnosis and new treatments have greatly lowered the mortality rate of young (aged 20–39 years) patients with cancer, and the overall all-cancer incidence and mortality rates in the USA showed a clear downward trend, declining more rapidly in males (16% and 21%, respectively) than females (2% and 17%) (Feng *et al.*, 2019). Despite a slight increase in the incidence rate in the UK from 2000 to 2012, the overall all-cancer mortality rate has declined by 11% in females and 15% in males (Feng *et al.*, 2019). Evidence suggests that most cancer survivors younger than 40 years expect their fertility to be maintained or their endocrine function to be restored (Donnez *et al.*, 2013). Sperm cryopreservation remains the only method to effectively preserve the reproductive potential of adult or adolescent male patients with cancer (Barak, 2019). The study of Huyghe showed that the fertility in patients with testicular cancer decreased by 30% after treatments: only 67.1% of patients get their partners pregnant successfully (Huyghe *et al.*, 2004). Data for cancer survivors failing to father children was limited; however, men with a history of cancer had two to three times higher odds of using IVF/ICSI treatments to father a child compared to those not being treated for cancer (Kitlinski *et al.*, 2023). One study reported that the chance of obtaining fatherhood was statistically significantly decreased after chemotherapy for testicular germ cell cancer, and the risk of needing ART to achieve fatherhood was increased after all treatment modalities (Bandak *et al.*, 2022). Moreover, it should be noted that clinical practice for fertility preservation recommendations and oncological treatment strategies has changed over the years. Unfortunately, we saw that, despite these changes, the numbers of cryopreservation procedures in cancer patients remained low. Meanwhile, surveys indicate a limited knowledge of guidelines and poor compliance with recommendations by medical professionals (Tholeti *et al.*, 2021; Huang *et al.*, 2022). Many patients also express dissatisfaction with the information provided by healthcare professionals regarding fertility risks and available options. Nevertheless, a significant obstacle that hampers the implementation of ideal fertility preservation practices is the absence of a structured and co-ordinated program (Brannigan *et al.*, 2021; Chen *et al.*, 2023). Some institutions have successfully implemented formal fertility preservation programs, which has led to a rise in the number of patients receiving fertility preservation consultations and using sperm cryopreservation services (Behl *et al.*, 2021).

The cancer types among male patients requiring fertility preservation include, for example, testicular germ cell tumors, lymphoma, leukemia, hematological neoplasms, and head and neck cancers. Cancer of the prostate (29%), lung and bronchus (12%), and colorectal (8%) were the most common forms of cancer diagnosed in men in 2023, while the greatest number of deaths were caused by lung (21%), prostate (11%), and colorectal (9%) cancer in male patients. The risk of developing invasive cancer varies significantly among different age groups, with the highest incidence in individuals aged 70 years or older. Furthermore, the rate of cancer among individuals from birth to 49 years is about 3.5%, including 0.4% for colorectal cancer, 0.3% for kidney cancer, 0.3% for lymphoma, 1% for lung cancer, 0.4% for skin melanoma, 0.3% for non-Hodgkin lymphoma, 0.2% for prostate cancer, and 0.2% for thyroid cancer (Siegel *et al.*, 2023). The highest survival rate is seen for cancers of the thyroid (98%), prostate (97%), testis (95%), and melanoma (94%), while the lowest survival rate is seen for cancers of the pancreas (12%), liver, and esophagus (21%) (Siegel *et al.*, 2023). Almost all studies in this meta-analysis included testicular germ cell tumors, Hodgkin’s or non-Hodgkin’s lymphoma, and leukemia because early-onset age and a good prognosis are the most common feature of these tumors (Pulte *et al.*, 2009; Ghazarian and McGlynn, 2020). Consequently, patients with these tumors will be concerned about their future fertility capacity. Negative effects on sperm quality, leading to defective spermatogenesis, are common in testicular malignancies (van Casteren *et al.*, 2010) and Hodgkin’s lymphoma (Paoli *et al.*, 2016). There is evidence that gonadotoxic treatment results in a significant reduction in sperm quality. The type of cancer and the pre-treatment sperm concentrations were found to be the most significant factors governing post-treatment semen quality and recovery of spermatogenesis (Bahadur *et al.*, 2005). Sperm quality varies with the type of underlying disease, with patients with testicular malignancy and hematological malignancies showing the lowest sperm counts (Vomstein *et al.*, 2021). However, some studies have not observed differences in sperm parameters among cancer patients (Auger *et al.*, 2016; Song *et al.*, 2019; Liu *et al.*, 2021). In the present study, failed-to-cryopreserve occurred in 10% (95% CI, 8–12%) of the patients. One of the most important causes was azoospermia, and it is important to consider this factor in future cost–benefit analysis. While we are still uncertain about the specific type of cancer affecting sperm quality, it is important to consider the potential impact of certain oncological treatments on the rate of azoospermia. The impact of cancer treatment on fertility is contingent upon the age of the patient at the time of diagnosis and treatment, as well as the type, duration, and dose intensity of treatment. Chemotherapy based on alkylating agents is linked to a higher risk of infertility in patients. Azoospermia is associated with chemotherapy and radiation, and whether it is temporary or permanent depends on the type of treatment, with radiation and alkylating agents posing the greatest risk for long-term damage (Meistrich, 2013). It is critical for clinicians to communicate effectively with their patients to guarantee that they freeze sperm before cancer treatment. This could potentially result in an increased use of ART.

This was the first meta-analysis to evaluate an association between male fertility preservation by way of sperm cryopreservation and reproductive outcomes. In this systematic review and meta-analysis, the inclusion of a significantly larger number of papers (69 compared with 30) and patients (32 234 compared with 11 798) did not contribute to outcomes that were significantly different from previous reviews (Ferrari *et al.*, 2016). The rate of use of frozen sperm remained low (9% compared with the previously reported 7.5% (van Casteren *et al.*, 2008) and 8% (Ferrari *et al.*, 2016)). We also noted that previously, fertility preservation was mostly performed in North America and Europe but is now performed globally, except in Africa, suggesting that economic development level might be related to the use of fertility preservation. Consequently, an in-depth analysis is needed to investigate the economic cost of sperm cryopreservation in male patients with cancer. From an economic point of view, the costs of the freezing process, long-term maintenance in a biobank, and the subsequent (when needed) use in ART are not trivial. Furthermore, only one-quarter of patients in the present study discarded their banked sperm (23%, 95% CI, 16–30%), indicating that most patients do not rule out the possibility of using their frozen semen in the future. However, spermatogenesis may have resumed in some of the patients who discard their banked spermatozoa. Radiotherapy and chemotherapy affect sperm concentration, and irradiation also increases sperm DNA fragmentation, which might be sustained for up to 2 years after treatment, affecting fertilization rates even after spermatogenesis recovery (Stahl *et al.*, 2006). In fact, more patients seem to dispose of sperm for no specific reason. The elapsed time between sperm freezing and the follow-up assessment is a fundamental factor that determines the utilization rate. A higher frozen sperm use rate might be related to a longer follow-up. Indeed, short follow-up is insufficient to draw information on frozen sperm use in young cancer survivors. Hence, the low rate for use of frozen sperm might be misleading. The observed 9% frozen sperm use might underestimate the real use rate.

The success rate and safety of ART using semen frozen before and during cancer treatments are issues of the greatest concern for reproductive medicine clinicians and oncologists. The documented pregnancy and delivery rates using thawed sperm collected before cancer therapy ranges from 7.0% to 66.7%, and 2.3% to 50.0%, respectively. The pregnancy and delivery rates after ICSI were higher than for IVF and IUI in the present study. According to an early study, it took a median of three cycles to achieve pregnancy in ICSI, whereas eight cycles were required in IVF (Kelleher *et al.*, 2001). Similar pregnancy rates have been observed compared with non-cancer controls or with fresh sperm (Schmidt *et al.*, 2004; García *et al.*, 2015). Indeed, it was disappointing to find that the clinical pregnancy and delivery rates were lower than theoretically expected (Boulet *et al.*, 2015; Hu *et al.*, 2020). This discrepancy could be because the included studies cover a wide timespan, with publication dates ranging from 1985 to 2021 (36 years). After all, the first successful treatment with conventional IVF was in 1978 (Steptoe and Edwards, 1978). In many respects, ART is still an immature field in its early stages of development. Our analysis also showed that more recent publications reported higher pregnancy and delivery rates, which may not be unexpected owing to improvements in ART over the years.

Our study has yielded some interesting insights into the impact of different tumor types on pregnancy and delivery rates. Notably, the pregnancy and delivery rates of lymphoma patients were lower than those of other cancer patients in this study. Indeed, there are many different subtypes of lymphatic tumors, each subtype having its own behavior, rate of progression, and response to treatment. The diffuse large B-cell lymphoma (DLBCL) is the most prevalent subtype of lymphoma among male patients (Ekberg *et al.*, 2020). A study from Sweden revealed that the cumulative rate of childbirth showed a decrease in the 10-year follow-up period among patients diagnosed with DLBCL, whereas no difference in fertility was observed among those with other types of lymphoma (Entrop *et al.*, 2023). In fact, different tumor types have distinct subtypes, and it may be biased to generalize the findings by pooling data from patients with heterogeneous tumor types, as these generalizations may not hold true for all tumor populations. Furthermore, this difference also may result from various ART approaches. Some studies have also reported decreased sperm counts and anti-Müllerian hormone concentration among lymphoma patients prior to treatment, suggesting that spermatogenesis was also affected by the disease (Pallotti *et al.*, 2021; Drechsel *et al.*, 2023).

The results of our meta-analysis also revealed that the pooled miscarriage rate after using frozen sperm was 13% (95%, CI, 10–17%) and, as ∼15–20% of all clinically confirmed pregnancies end in a miscarriage (Hooker *et al.*, 2014), our data align with data from the general population. A critical indicator of miscarriage is the absence of fetal heart activity. However, most included studies did not provide data on fetal heart activity. It is important to note that a single ultrasound indicating the absence of a fetal heartbeat is not immediately conclusive. Depending on the circumstances and the specific timing during the pregnancy, waiting and performing another ultrasound a week later to verify the absence of fetal heart activity before definitively diagnosing a miscarriage is recommended. Therefore, the absence of data on fetal heart activity may introduce potential bias in the miscarriage data.

In fact, ART plays a key role in future fertility planning of patients with cancer aiming for fertility preservation. However, it should be noted that there is heterogeneity among human sperm banking services based on geographical location or setting. The decision of patients to bank or use sperm, as well as the follow-up rate, may be influenced by national legislation or requirements. For example, the availability and coverage of costs for banking and IVF procedures could be influenced by the national health system or insurance companies. Additionally, local guidelines play a role, such as whether fertility centers regularly follow-up with patients. These variations can further impact the interpretation of data regarding the success rate and rate of use of frozen sperm.

As with all meta-analyses, some limitations should be considered. The first limitation of our study is that the included data span 36 years. During this time, the WHO has revised its sperm analysis standards, and other important changes have been seen, including improved cancer survival rates, modifications of cancer treatment regimens, increased incidence of malignancies during reproductive age, and increased awareness among oncologists of the availability of sperm cryopreservation for male fertility preservation. Another limitation is that our aim was limited to the failed-to-cryopreserve rate, sperm disposal rate, sperm use rate, pregnancy rate, miscarriage rate, and delivery rate as the outcome of fertility preservation. However, it is important to note though, that these rates represent only some of the potential factors analyzed for the beneficial effects in male cancer patients and that these findings do not extend to an analysis of the impact of different types of cancer on sperm quality. Many of the earlier studies were limited by small sample sizes and a lack of control groups. Furthermore, almost all of studies did not consider the severity of the disease, which could potentially have a substantial impact on the results. Consequently, further research should evaluate the effect of the type of cancer and, in particular, the severity of the condition on sperm quality in order to draw more precise conclusions. Similarly, it is inappropriate that most studies failed to differentiate between types of tumors and, instead, drew generalized conclusions that are presumed to apply to all cancer patients. We did not have in-depth information on the patients’ disease and, although extensive efforts were made to conduct a thorough systematic review and meta-analysis of the outcomes in terms of the various tumor types, the results must be acknowledged as being subject to bias. On the other hand, the use of average results obtained in each included study, without the individual patient-level data, might also represent a source of bias. Finally, almost no studies provided in-depth information regarding the semen and its usage from the same patient after multiple ejaculations. Such information is valuable, as it can be used to customize the care model to the individual.

In conclusion, our study supported previous reports that sperm cryopreservation is an effective method of fertility preservation in male patients with cancer. The rate of use of frozen sperm in our review underestimated the actual rate, making it meaningful to actively recommend fertility preservation to patients with cancer. ART plays an important role in fertility preservation and pregnancy achievement, with ICSI resulting in better clinical outcomes than IVF and IUI in patients with cancer. Hence, fertility preservation still requires the involvement of oncologists, reproductive medicine clinicians, andrologists, and embryologists.

## Supplementary Material

hoae006_Supplementary_Figure_S1

hoae006_Supplementary_Figure_S2

hoae006_Supplementary_Figure_S3

hoae006_Supplementary_Figure_S4

hoae006_Supplementary_Table_S1

## Data Availability

Literature research results are available from the authors upon reasonable request.
