# Supplementary figures and images for: Fertility preservation in adult male patients with cancer: a systematic review and meta-analysis
Source: Hum Reprod Open. 2024 Jan 30;2024(1):hoae006. doi: 10.1093/hropen/hoae006 (PMC10882264; doi:10.1093/hropen/hoae006)

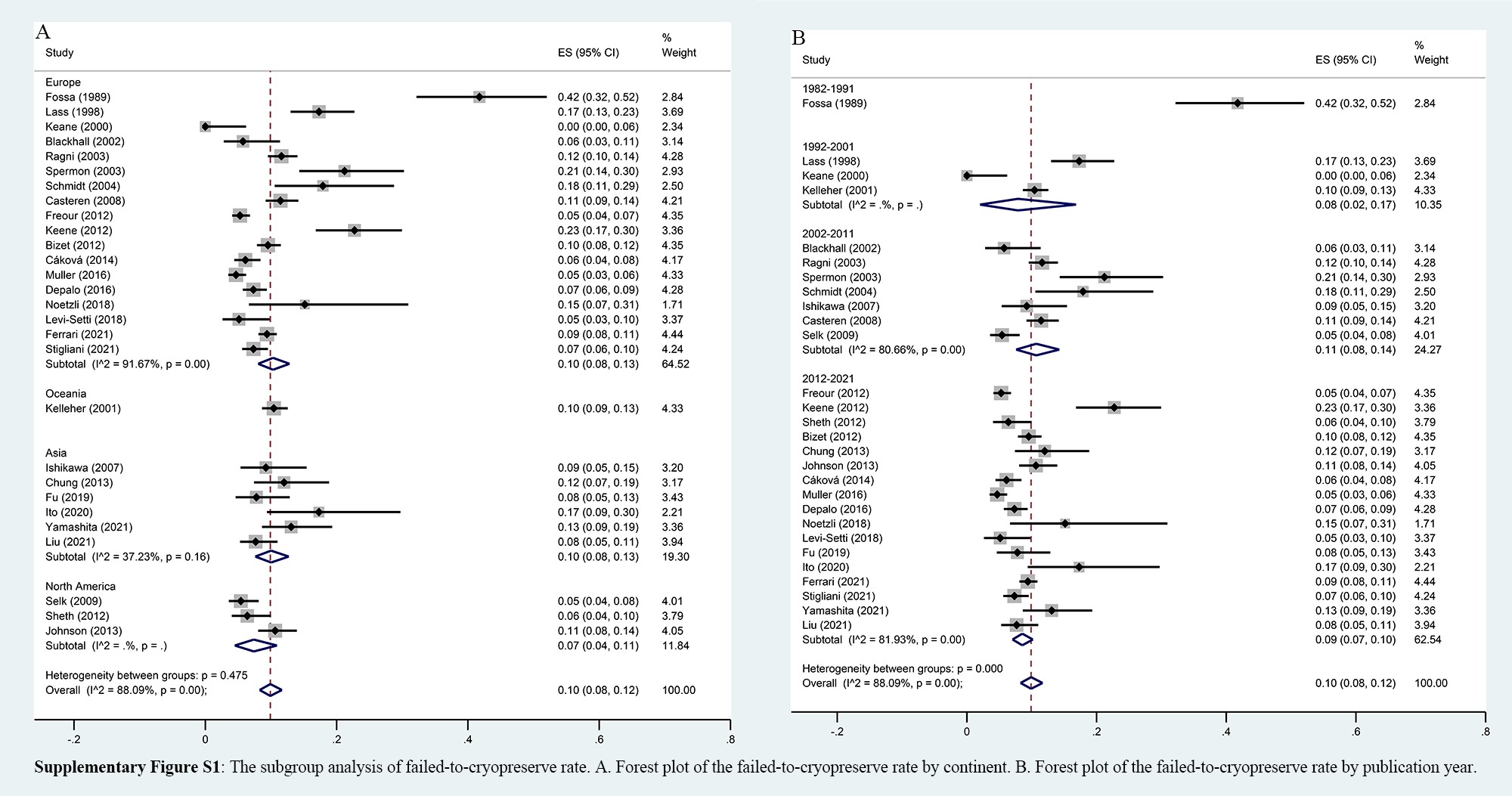

Supplement: hoae006_Supplementary_Figure_S1 [file hoae006_supplementary_figure_s1.jpeg]

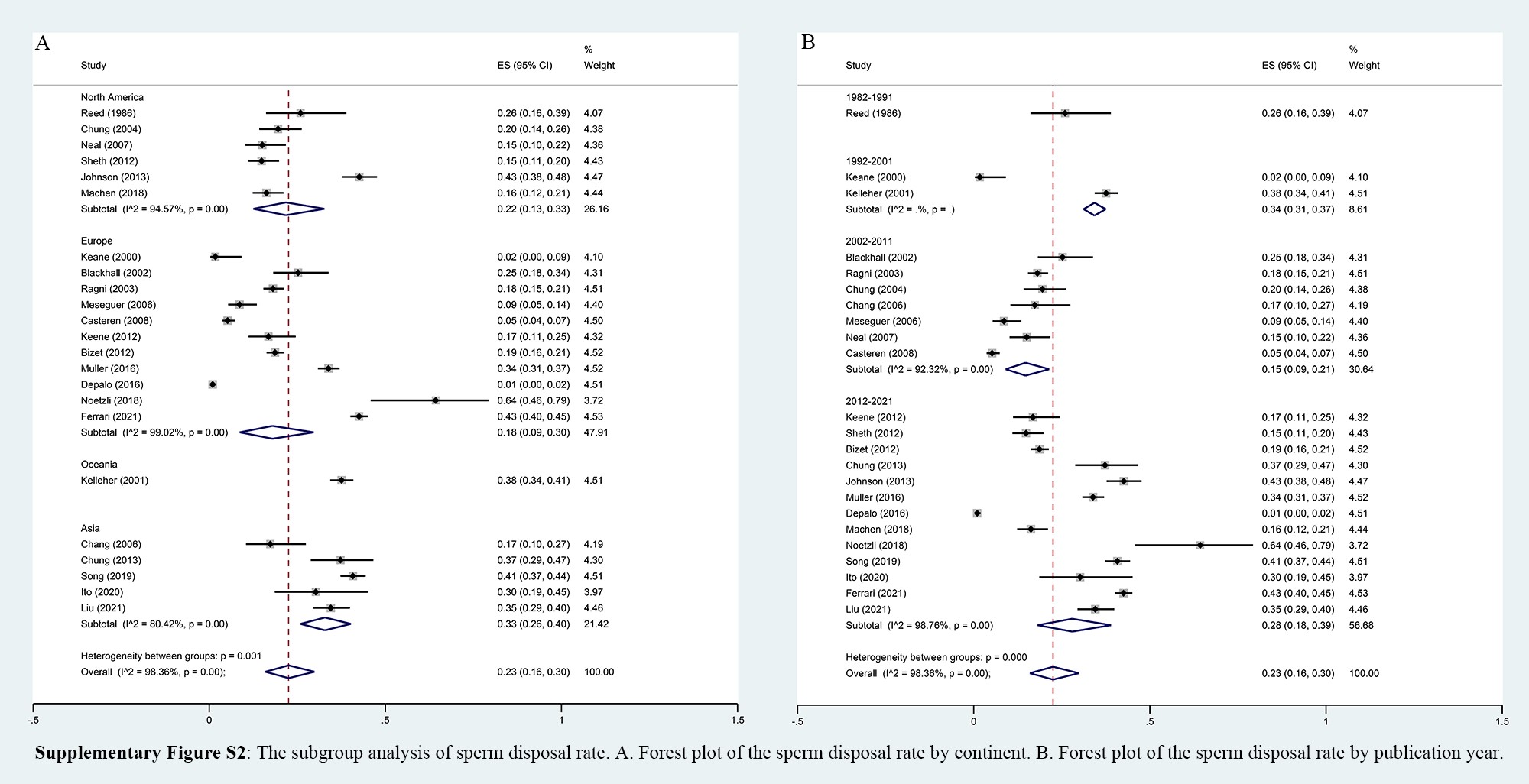

Supplement: hoae006_Supplementary_Figure_S2 [file hoae006_supplementary_figure_s2.jpeg]

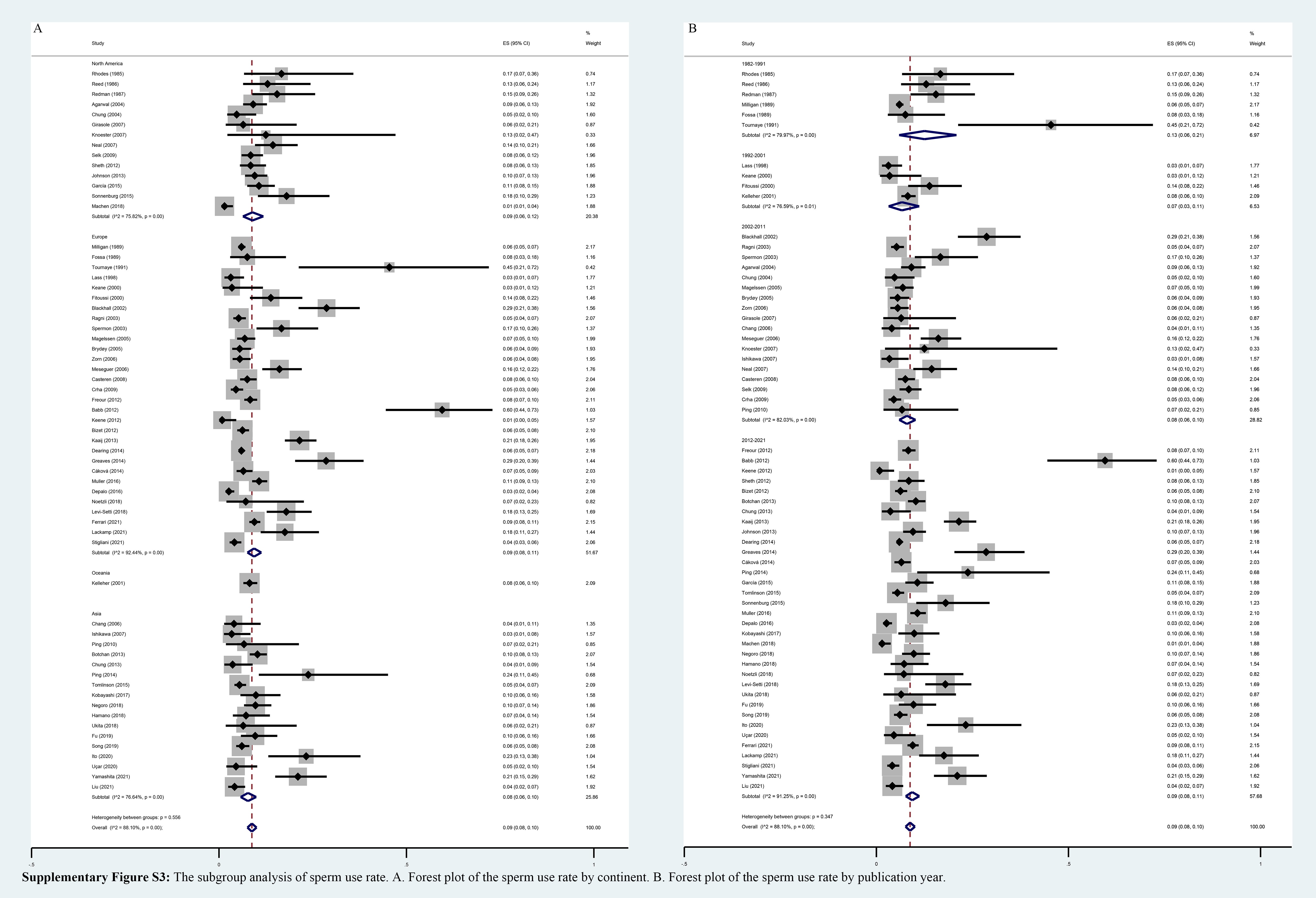

Supplement: hoae006_Supplementary_Figure_S3 [file hoae006_supplementary_figure_s3.jpeg]

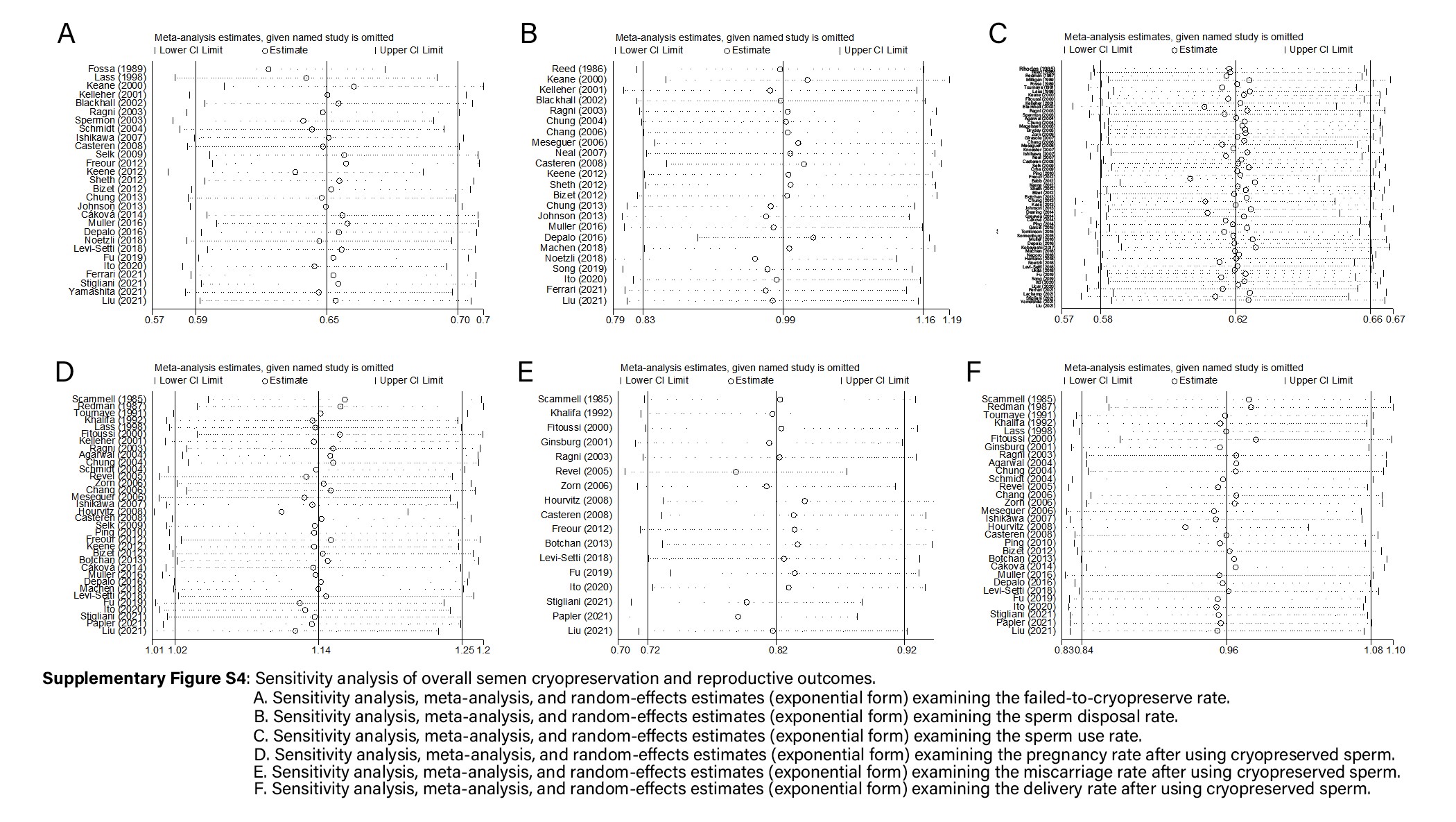

Supplement: hoae006_Supplementary_Figure_S4 [file hoae006_supplementary_figure_s4.jpeg]
