# Supplementary material for: Fertility preservation in adult male patients with cancer: a systematic review and meta-analysis
Source: Hum Reprod Open. 2024 Jan 30;2024(1):hoae006. doi: 10.1093/hropen/hoae006 (PMC10882264; doi:10.1093/hropen/hoae006)
Supplement: hoae006_Supplementary_Table_S1 [file hoae006_supplementary_table_s1.docx]

**Supplementary Table S1.** Quality assessment of studies according to the Newcastle-Ottawa quality assessment scale and Methodological Index for Non-Randomized Studies.

| Author | 1. A clearly stated aim | 2. A clearly defined study population | 3. Representativeness of the sample | 4. Report of excluded patients | 5. Ascertainment of exposure | 6. Prospective collection of data | 7. Presence of the outcome of interest | 8. Adequate assessment of the outcome of interest | 9. Loss to follow-up | 10. Prospective calculation of the study size | Quality score |
| --- | --- | --- | --- | --- | --- | --- | --- | --- | --- | --- | --- |
| Rhodes *et al.,*1985 | 1 | 0 | 1 | 0 | 1 | 0 | 0 | 1 | 1 | 0 | 5 |
| Scammell *et al.,*1985 | 1 | 0 | 1 | 0 | 1 | 0 | 0 | 1 | 0 | 0 | 4 |
| Reed *et al.,*1986 | 1 | 0 | 1 | 0 | 1 | 0 | 0 | 1 | 0 | 0 | 4 |
| Redman *et al.,*1987 | 1 | 0 | 1 | 0 | 1 | 0 | 1 | 1 | 1 | 0 | 6 |
| Milligan *et al.,*1989 | 1 | 0 | 1 | 0 | 1 | 0 | 1 | 0 | 0 | 0 | 4 |
| Fossa *et al.,*1989 | 1 | 1 | 1 | 1 | 1 | 0 | 1 | 1 | 0 | 0 | 7 |
| Tournaye *et al.,*1991 | 1 | 0 | 1 | 0 | 1 | 0 | 1 | 0 | 0 | 0 | 4 |
| Khalifa *et al.,1*992 | 1 | 1 | 1 | 0 | 1 | 0 | 1 | 1 | 0 | 0 | 6 |
| Lass *et al.,*1998 | 1 | 1 | 1 | 0 | 1 | 0 | 1 | 0 | 0 | 0 | 5 |
| Keane *et al.,*2000 | 0 | 1 | 1 | 0 | 1 | 0 | 1 | 0 | 0 | 0 | 4 |
| Fitoussi *et al.,*2000 | 1 | 1 | 1 | 0 | 1 | 0 | 1 | 1 | 0 | 0 | 6 |
| Ginsburg *et al.,*2001 | 1 | 1 | 1 | 1 | 1 | 0 | 1 | 1 | 0 | 0 | 7 |
| Kelleher *et al.,*2001 | 1 | 1 | 1 | 0 | 1 | 1 | 1 | 1 | 0 | 0 | 7 |
| Blackhall *et al.,*2002 | 1 | 1 | 1 | 1 | 1 | 0 | 1 | 1 | 0 | 0 | 7 |
| Ragni *et al.,*2003 | 1 | 1 | 1 | 1 | 1 | 0 | 1 | 1 | 0 | 0 | 7 |
| Spermon *et al.,*2003 | 1 | 1 | 1 | 1 | 1 | 0 | 1 | 1 | 0 | 0 | 7 |
| Agarwal *et al.,*2004 | 1 | 1 | 1 | 0 | 1 | 1 | 1 | 1 | 0 | 0 | 7 |
| Chung *et al.,*2004 | 1 | 1 | 1 | 0 | 1 | 0 | 1 | 1 | 0 | 0 | 7 |
| Schmidt *et al.,*2004 | 1 | 1 | 1 | 0 | 1 | 0 | 1 | 1 | 0 | 0 | 6 |
| Revel *et al.,*2005 | 1 | 1 | 1 | 0 | 1 | 0 | 1 | 1 | 0 | 0 | 6 |
| Zorn *et al.,*2006 | 1 | 1 | 1 | 0 | 1 | 0 | 1 | 1 | 0 | 0 | 6 |
| Magelssen *et al.,*2005 | 1 | 1 | 1 | 1 | 1 | 0 | 1 | 1 | 0 | 0 | 7 |
| Brydøy *et al.,*2005 | 1 | 1 | 1 | 1 | 1 | 0 | 1 | 1 | 1 | 0 | 8 |
| Girasole *et al.,*2007 | 1 | 1 | 1 | 0 | 1 | 0 | 1 | 1 | 0 | 0 | 6 |
| Chang *et al.,*2006 | 1 | 1 | 1 | 0 | 1 | 0 | 1 | 1 | 0 | 0 | 6 |
| Meseguer *et al.,*2006 | 1 | 1 | 1 | 1 | 1 | 1 | 1 | 1 | 1 | 0 | 9 |
| 2007 Knoester | 1 | 1 | 1 | 0 | 1 | 0 | 1 | 0 | 0 | 0 | 5 |
| Ishikawa *et al.,*2007 | 1 | 1 | 1 | 0 | 1 | 0 | 1 | 1 | 0 | 0 | 6 |
| Neal *et al.,*2007 | 1 | 1 | 1 | 1 | 1 | 0 | 1 | 1 | 1 | 0 | 8 |
| Hourvitz *et al.,*2008 | 1 | 1 | 1 | 0 | 1 | 0 | 1 | 1 | 0 | 0 | 6 |
| Casteren *et al.,*2008 | 1 | 1 | 1 | 1 | 1 | 0 | 1 | 1 | 1 | 0 | 8 |
| Selk *et al.,*2009 | 1 | 1 | 1 | 0 | 1 | 0 | 1 | 1 | 0 | 0 | 6 |
| Crha *et al.,*2009 | 1 | 1 | 1 | 0 | 1 | 1 | 1 | 1 | 0 | 0 | 7 |
| Ping *et al.,*2010 | 1 | 1 | 1 | 0 | 1 | 0 | 1 | 1 | 0 | 0 | 6 |
| Freour *et al.,*2012 | 1 | 1 | 1 | 0 | 1 | 0 | 1 | 1 | 0 | 0 | 6 |
| Babb *et al.,*2012 | 1 | 1 | 1 | 1 | 1 | 0 | 1 | 1 | 0 | 0 | 7 |
| Keene *et al.,*2012 | 1 | 1 | 1 | 1 | 1 | 0 | 1 | 1 | 0 | 0 | 7 |
| Sheth *et al.,*2012 | 1 | 1 | 1 | 1 | 1 | 0 | 1 | 1 | 1 | 0 | 8 |
| Bizet *et al.,*2012 | 1 | 1 | 1 | 1 | 1 | 0 | 1 | 1 | 0 | 0 | 7 |
| Botchan *et al.,*2013 | 1 | 1 | 1 | 1 | 1 | 0 | 1 | 1 | 1 | 0 | 8 |
| Chung *et al.,*2013 | 1 | 1 | 1 | 0 | 1 | 0 | 1 | 1 | 1 | 0 | 7 |
| Kaaij *et al.,*2013 | 1 | 1 | 1 | 1 | 1 | 1 | 1 | 1 | 1 | 0 | 9 |
| Johnson *et al.,*2013 | 1 | 1 | 1 | 1 | 1 | 0 | 1 | 1 | 1 | 0 | 8 |
| Dearing *et al.,*2014 | 1 | 1 | 1 | 0 | 1 | 0 | 1 | 1 | 1 | 0 | 7 |
| Greaves *et al.,*2014 | 1 | 1 | 1 | 1 | 1 | 0 | 1 | 1 | 1 | 0 | 8 |
| Cáková *et al.,*2014 | 1 | 1 | 1 | 0 | 1 | 0 | 1 | 1 | 0 | 0 | 6 |
| Ping *et al.,*2014 | 1 | 1 | 1 | 1 | 1 | 0 | 1 | 1 | 1 | 0 | 8 |
| García *et al.,*2015 | 1 | 1 | 1 | 0 | 1 | 0 | 1 | 1 | 1 | 0 | 7 |
| Tomlinson *et al.,*2015 | 1 | 1 | 1 | 0 | 1 | 0 | 1 | 1 | 0 | 0 | 6 |
| Sonnenburg *et al.,*2015 | 1 | 1 | 1 | 1 | 1 | 0 | 1 | 0 | 1 | 0 | 7 |
| Muller *et al.,*2016 | 1 | 1 | 1 | 1 | 1 | 0 | 1 | 1 | 1 | 0 | 8 |
| Depalo *et al.,*2016 | 1 | 1 | 1 | 1 | 1 | 0 | 1 | 1 | 1 | 0 | 8 |
| Kobayashi *et al.,*2017 | 1 | 1 | 1 | 1 | 1 | 0 | 1 | 1 | 0 | 0 | 7 |
| Machen *et al.,*2018 | 1 | 0 | 1 | 0 | 1 | 0 | 1 | 1 | 0 | 0 | 5 |
| Negoro *et al.,*2018 | 1 | 1 | 1 | 1 | 1 | 0 | 1 | 1 | 0 | 0 | 7 |
| Hamano *et al.,*2018 | 1 | 1 | 1 | 1 | 1 | 0 | 1 | 1 | 1 | 0 | 8 |
| Noetzli *et al.,*2018 | 1 | 1 | 1 | 1 | 1 | 0 | 1 | 1 | 1 | 0 | 8 |
| Levi-Setti *et al.,*2018 | 1 | 1 | 0 | 1 | 1 | 0 | 1 | 1 | 1 | 0 | 7 |
| Ukita *et al.,*2018 | 1 | 1 | 1 | 0 | 1 | 0 | 1 | 1 | 0 | 0 | 6 |
| Fu et al.,2019 | 1 | 1 | 1 | 1 | 1 | 0 | 1 | 1 | 1 | 0 | 8 |
| Song *et al.,*2019 | 1 | 1 | 1 | 1 | 1 | 0 | 1 | 1 | 1 | 0 | 8 |
| Ito *et al.,*2020 | 1 | 1 | 1 | 1 | 1 | 0 | 1 | 1 | 1 | 0 | 8 |
| Uçar *et al.,*2020 | 1 | 1 | 1 | 0 | 1 | 0 | 1 | 1 | 0 | 0 | 6 |
| Ferrari *et al.,*2021 | 1 | 1 | 1 | 1 | 1 | 0 | 1 | 1 | 1 | 0 | 8 |
| Lackamp *et al.,*2021 | 1 | 1 | 1 | 0 | 1 | 0 | 1 | 1 | 0 | 0 | 6 |
| Stigliani *et al.,*2021 | 1 | 1 | 1 | 1 | 1 | 0 | 1 | 1 | 1 | 0 | 8 |
| Yamashita *et al.,*2021 | 1 | 1 | 1 | 0 | 1 | 0 | 1 | 0 | 0 | 0 | 5 |
| Papler *et al.,*2021 | 1 | 1 | 1 | 0 | 1 | 0 | 1 | 1 | 0 | 0 | 6 |
| Liu *et al.,*2021 | 1 | 1 | 1 | 1 | 1 | 0 | 1 | 1 | 1 | 0 | 8 |
